# Gene Expression Status and Methylation Pattern in Promoter of P15INK4b and P16INK4a in Cord Blood CD34^**+**^ Stem Cells 

**Published:** 2013-07

**Authors:** Mehdi Azad, Saeid Kaviani, Mehrdad Noruzinia, Yousef Mortazavi, Naser Mobarra, Shaban Alizadeh, Mohammad Shahjahani, Fatemeh Skandari, Mohammad Hosein Ahmadi, Amir Atashi, Saeid Abroun, Zahra Zonoubi

**Affiliations:** 1 Hematology Department, School of Medical Sciences, Tarbiat Modares University, Tehran, Iran; 2 Hematology Department, Zanjan Medical Sciences University, Zanjan, Iran; 3 Department of Clinical Biochemistry, School of Medicine, Tehran University of Medical Sciences, Tehran, Iran; 4 Department of Hematology, Allied Medical School, Tehran University of Medical Sciences, Tehran, Iran; 5 Iranian Blood Transfusion Organizations, Medical Department; 6 Department of Obstetrics and Gynecology, Mahdiyeh Hospital, Shahid Beheshti University,Tehran, Iran; 7 Students’ Scientiﬁc Research Center, Tehran University of Medical Sciences, Tehran, Iran

**Keywords:** Gene expression, Hematopoietic stem cells, Methylation, Tumor suppressor genes

## Abstract

***Objective(s)*** ***:*** Stem cell differentiation into different cell lineages depends upon several factors, cell cycle control elements and intracellular signaling elements, including P15INK4b and P16INK4a genes. Epigenetics may be regarded as a control mechanism which is affected by these factors with respect to their promoter structure.

***Materials and Methods*** ***: ***The CD34 + cord blood stem cells were purified, isolated and then expanded. The undifferentiated day genome was isolated from part of the cultured cells, and the seventh day differentiated genome was isolated from the other part after differentiation to erythroid lineage. The procedure was followed by a separate Real-Time PCR for the two genes using the obtained cDNA. The processed DNA of the former stages was used for MSP (Methylation Specific PCR) reaction. Finally, pre- and post differentiation results were compared.

***Results*** ***:*** After performing MSP for each gene, it became clear that P15INK4b gene has undergone methylation and expression in predifferentiation stage. In addition, its status has not been changed after differentiation. P15INK4b gene expression was reduced after the differentiation. The other gene, P16INK4a, showed no predifferentiation methylation. Itwas completely expressed methylated and underwent reduced expression after differentiation.

***Conclusion*** **:** Specific predifferentiation expression of P15INK4b and P16INK4a genes along with reduction in their expression after erythroid differentiation indicated animportant role for these two genes in biology of CD34+ cells in primary stages and before differentiation. In addition, both genes are capable of epigenetic modifications due to the structure of their promoters.

## Introduction

Investigations about the formation mechanisms of different cell types *in vitro* has led to understanding of the general mechanisms of transcription and regulation of the gene expression (1-3). In fact, differentiation process in primitive cells depends on the control of gene expression and precise regulation of the intracellular signaling. In this context, the requirement of specific control factors such as cytokines, specific transcription factors and cell cycle control elements is established and may be expected (4-8). Two of these factors classified as tumor suppressor proteins include cyclin-dependent kinase inhibitors of 2A and 2B types, respectively, known as P16INK4a and P15INK4b. P16INK4a or P16 is a tumor suppressor protein and cell cycle control element. A study by Minami *et al* (2003) highlighted the role of P16 in hematopoiesis. They showed that this gene was able to induce differentiation and apoptosis in erythroid lineage (9). CDK-4 and CDK6 are potentially inhibited by P16, therefore, the mdm 2 is not activated, and P53 protein (degraded in normal conditions by mdm 2) remains intact in these conditions and may suppress the activity of tumors (9-12). The genes related to P15 and P16 are adjacent to each other on the same locus, and therefore their deletion in most tumors occurs simultaneously. P15INK4b or P15 gene is also a tumor suppressor protein. This gene inhibits complex formation of cyclin D with CDK4 and CDK6 kinases, and thus controls the cell cycle at G1 point (10-13). Epigenetics is an important topic introduced as one of the pathways controlling gene expression, defined as the changes in gene expression without any basic changes in sequences. Recently, epigenetics has been addressed as a principle in the regulation of tissue differentiation, capable of significantly altering all stages in the growth and proliferation of cells according to a broad range of previous studies. An important and effective epigenetic mechanism is methylation of promoter regions of genes resulting in quenching of the respective genes (14-20). In addition, many studies have investigated the genesis and evolution processes of the cell, hence, various mechanisms including epigenetics have been proposed. An important component of these mechanisms is tumor suppressor factors, two examples of which have been evaluated in the present study (1-8). These factors play an important role in controlling and changing the genetic profile of terminally differentiated cells, with methylation considered as a control mechanism of the gene profile (18-20). The methylation pattern and P15 and P16 expression status are effective factors in controlling cell proliferation and differentiation, which have not been comparatively evaluated so far in CD_34_^+ ^cells before and after erythroid differentiation. In this study we have addressed this issue and compared the expression patterns of the mentioned genes along with their methylation status in purified stem cells from cord blood in pre and post differentiation stages. 

## Materials and Methods

***Isolation and*** ***expansion of CD34 +*** ***stem cells***

Cord blood bags from Sarem Hospital and Iranian Blood Transfusion Organization, Tehran, Iran were collected and their CD34+ cells were purified using indirect CD34 MicroBead Kit of Biotec Miltenyi Company using MACS method. Afterwards, using Stem Span medium enriched by growth factors Flt3, TPO and SCF andthe isolated cells were expanded and divided into two parts after washing with the PBS buffer. The first part was used for DNA and RNA extractions related to the predifferentiation stage. 

***Determining the purity of hematopoietic cells after the amplification process***

This step was performed using the monoclonal anti-CD34 antibody and phycoerythrin as fluorescent dye.

***Differentiation*** ***to erythroid lineage ***

The second part of the expanded cells was used for induction of erythroid differentiation. Afterwards, the miR-451 precursor was used, the role of which has been demonstrated previously in erythroid differentiation of stem cells (21, 22). Before adding miR-451 and Lipofectamine to the desired differentiation medium, the appropriate amount of OPTI-MEM medium was added and set at room temperature for a few minutes. In the next step, the factors, prepared in 24 well culture plates, were added to the IMDM + FBS 10% + SCF differentiation medium and incubated for 48 hours. The miR transfer process was conducted every other day as mentioned for three consecutive times, and in the seventh day of differentiation, erythroid precursors were used for purification of the differentiated DNA. 

***Erythroid differentiation confirmation***

At this stage, using the monoclonal antibody against erythroid lineage differentiation indicator of CD71 and seventh day differentiated cells, flow cytometric analysis was performed. Finally, for further confirmation, PCR reaction was performed using primers for erythroid differentiation markers (CD235 and CD71) along with differentiated and undifferentiated cDNA.

***Isolation of DNA and RNA from the expanded cells***

Pre- and post differentiation DNA and RNA were purified using DNA and RNA extraction kits from Qiagen Company. The extracted DNA and RNA were put in a -20°C freezer for a short period of time for subsequent tests.

***Processing*** ***DNA*** ***and*** ***RNA*** ***for*** ***MSP*** ***and*** ***Real-Time PCR***

At this stage, DNA stored from the previous stages was processed using EpiTect® Bisulfite kit and transferred to a -20°C freezer. SssI was used to prepare positive control for MSP technique. Subsequently, cDNA synthesis was performed using Qiagen kit and the stored RNA in previous steps, and was stored in a -20°C freezer. 

***Gene expression*** ***study using Real-Time PCR ***

Using differentiated and undifferentiated cDNA of day seven, Real-Time PCR was conducted separately for both genes, and the data were interpreted using Pfaffl calculations. At this stage, GAPDH was employed to control the genes. ABI 7500 instrument was used for Real-Time PCR with SYBR green as the Master Mix. The sequence of the primers has been shown in Table 1.

**Table 1:** Profile of primers used in Real-Time PCR

Oligo name	5′ to 3′ Sequence
P15-F	GGGAAAGAAGGGAAGAGTGTCGTT
P15-R	GCATGCCCTTGTTCTCCTCG
P16-F	GGGGGACCAGAGGCAGT
P16-R	GGTTGTGGCGGGGGCAGTT

**Table 2:** Profile of primers used in MSP

Gene	Size (bp)	Cycles	Temp ^O^C	Form	5^′^ to 3^′^ Sequences
P15	249	38	50	UF	TGT GAT GTG TTT GTA TTT TGT GGT T
				UR	CCA TAC AAT AAC CAA ACA ACC AA
	237	38	60	MF	GGTTC GTA TTT TGC GGT T
				MR	CGT ACA ATA ACC GAA CGA CCG A
P16	151	35	60	UF	TTA TTA GAG GGT GGG GTG GAT TGT
				UR	CAA CCC CAA ACC ACA ACC ATA A
	150	35	65	MF	TTA TTA GAG GGT TCT GAT CGC
				MR	GAC CCC GAA CCG CGA CCG TAA

***MSP*** ***reaction*** ***to assess the methylation pattern***

Next, using differentiated and undifferentiated DNA, MSP was conducted for each gene separately. Details of primers used in this step are listed in Table 2. 

***Statistical analysis***

All experiments were repeated three times and data were presented as the mean ± SD. The comparison between groups was performed by Student's t-test. A* P-*value of less than 0.05 considered as a statistically significant. 

## Results

**Figure 1:**
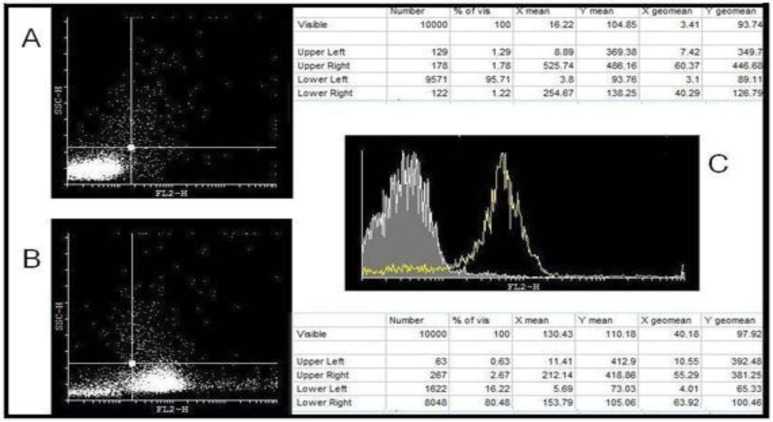
CD34 marker expression on undifferentiated cells on day zero following the amplification process

Flow cytometry results for the purity of cells isolated from umbilical cord blood after amplification are exhibited in Figure 1. These results were obtained using anti-CD34 monoclonal antibody and BD flow cytometry system. Phycoerythrin was used as a fluorescent dye for labeling the anti-CD34 antibody in this step. Cyflogic Software was used to interpret the data. Figure A-1 relates to isotype control and Figure B-1concerns CD34. Figure C-1 indicates 80% purity rate in the cells isolated from cord blood following the expansion. 

**Figure 2:**
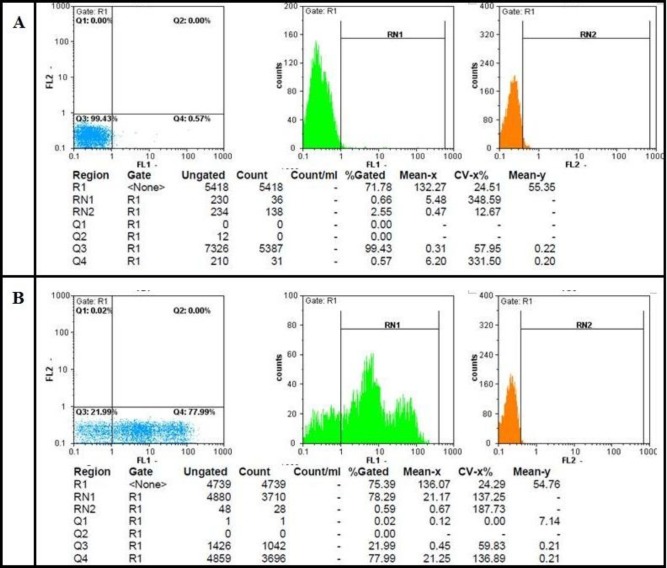
The transferrin receptor (CD71) expression on the cells of seventh day differentiation using Partec flow cytometer. Figure 2-A is related to isotype control and figure 2-B to CD71

**Figure 3:**
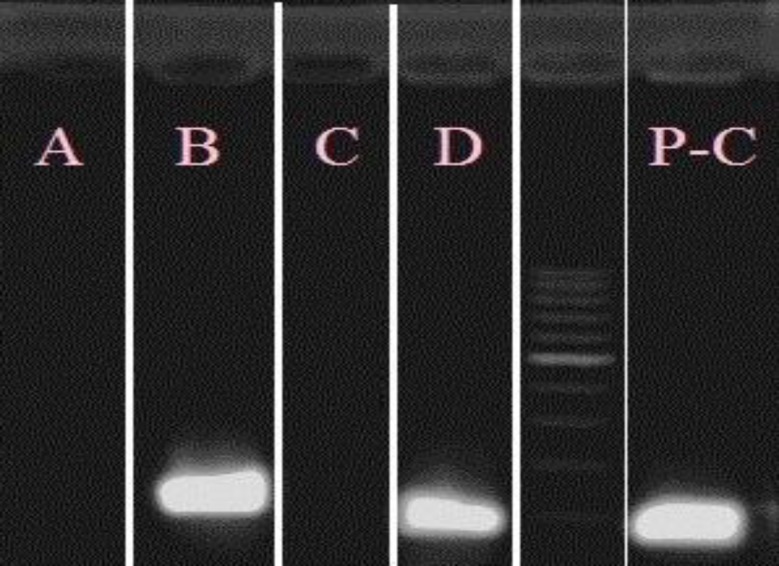
The well A is related to the undifferentiated cDNA and specific CD71 primers, and the well C also relates to this same type of cDNA and specific primers of CD235, and absence of band in both of them indicates no expression of CD71 and CD235 in predifferentiation stage. The B and D wells having specific bands relate to the differentiated cDNA of day seven. Product of the B well indicates a clear expression of CD71, and product of well D indicates a clear expression of CD235

As already mentioned, to confirm the differentiation of stem cells to erythroid lineage, flow cytometric analysis and PCR were performed. In fact, flow cytometric results with Anti-CD71 were quite remarkable and showed 78% rate of differentiation. Flow cytometric results are shown in Figure 2. On the other hand, the banding pattern of erythroid differentiation factors indicated an appropriate differentiation towards red cell precursors (Figure 3). 

**Figure 4:**
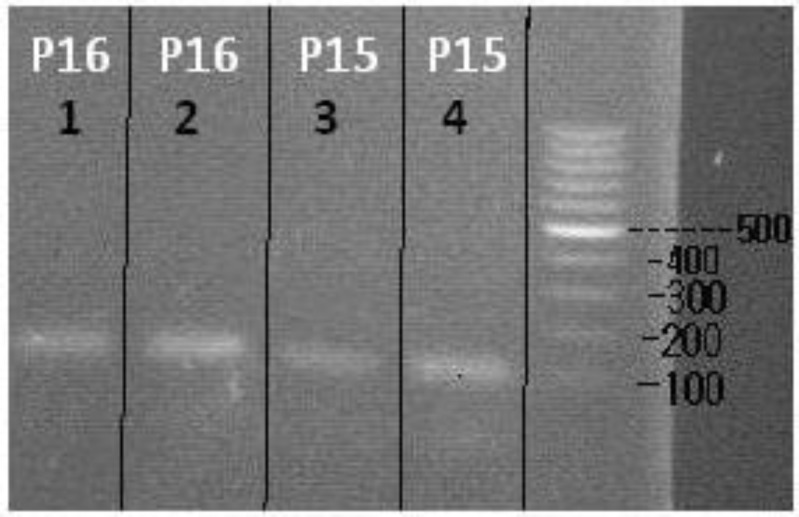
The above Figure is related to 1.5% agarose gel, in which the visible bands indicate PCR reaction products for the genes studied in this research. The wells 1 and 3 bands are related to undifferentiated cDNA, and bands of wells 2 and 4 are related to differentiated cDNA. According to the Figure, both factors under study in this research are more or less expressed in the stem cells under study

As mentioned, after the quality control and processing of the differentiated and undifferentiated genomes using Biophotometer instrument, the cDNA prepared from stem cells before and after differentiation was subject to the Real-Time PCR to quantitatively determine the expression of the genes. In addition, to ensure the presence of product, the sample obtained after Real-Time was set on 1.5% agarose gel, and evaluated using Gel Documentation instrument. Band length of DNA related to the PCR products for p15 and p16 genes were almost close together and were 104 bp and 159 bp, respectively (Figure 4).

**Figure 5:**
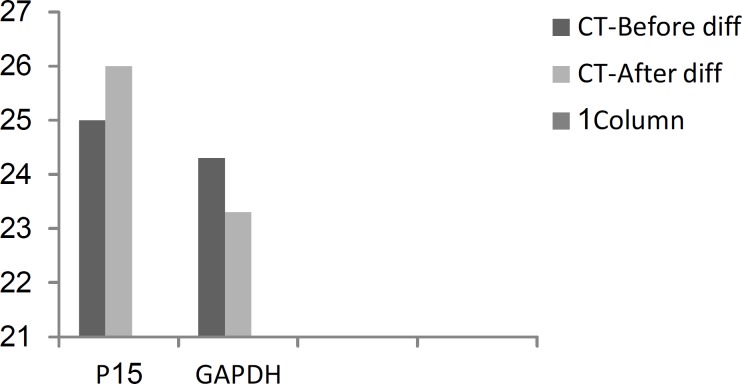
CT values from Real-Time PCR, for P15 gene and Control GAP, before and after the differentiation

Real-Time PCR results for P15 gene suggested reduced expression of this gene in post differentiation stage. In fact, the expression of P15 after erythroid differentiation was approximately 0.3 times of its expression level in predifferentiation stage. In other words, the predifferentiation expression level of the P15 is three times of the level of post differentiation. CT (Cycle Threshold) values related to P15 are displayed in Figure 5. 

**Figure 6:**
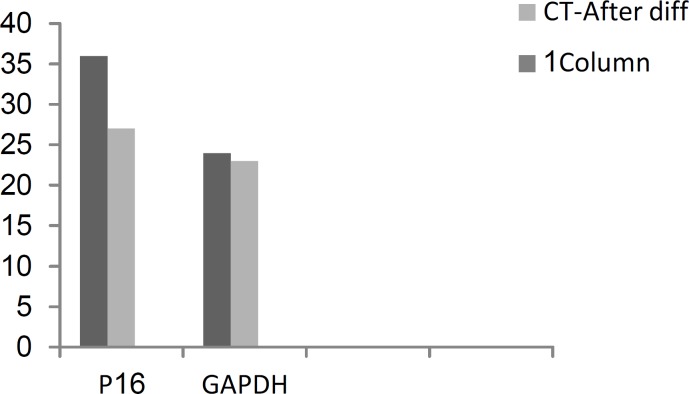
CT values from Real-Time PCR, for P16 Gene and Control GAP, before and after the differentiation

The results for P16 also showed reduced expression of this gene in post differentiation stage relative to the predifferentiation stage. In fact, the expression of P16 after differentiation was approximately 0.9 times of its expression level in predifferentiation stage. CT (Cycle Threshold) values related to P16 are shown in Figure 6.

**Figure 7:**
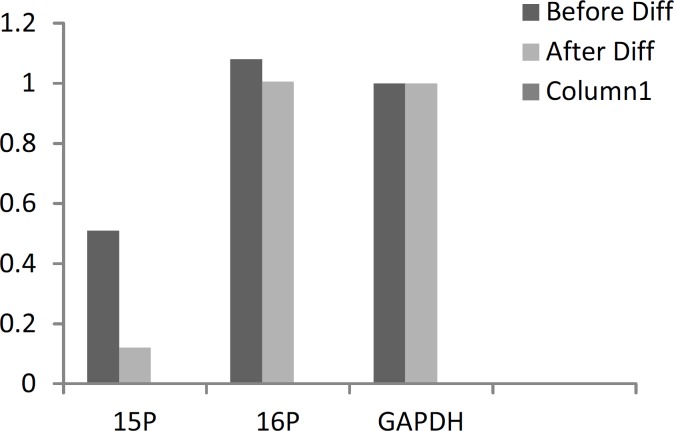
Normalized values of gene expression before and after the differentiation

**Figure 8:**
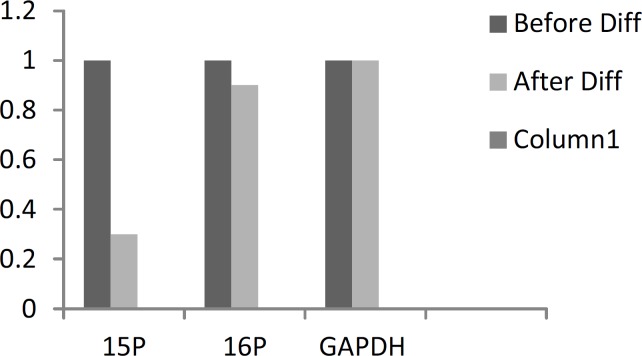
Calibrated ratio of gene expression (before to after the differentiation)

In Figures 7 and 8, the expression values of the genes under study are displayed after correction.

In another part of this study, DNA was subject to the MSP test after processing to assess the pattern of methylation. The DNA bands in MSP for the genes under study are as follows:

1. Methylated P16 Gene – 150 bp
2. Unmethylated P16 Gene – 151 bp
3. Methylated P15 Gene – 154 bp
4. Unmethylated P15 Gene – 148 bp

**Figure 9:**
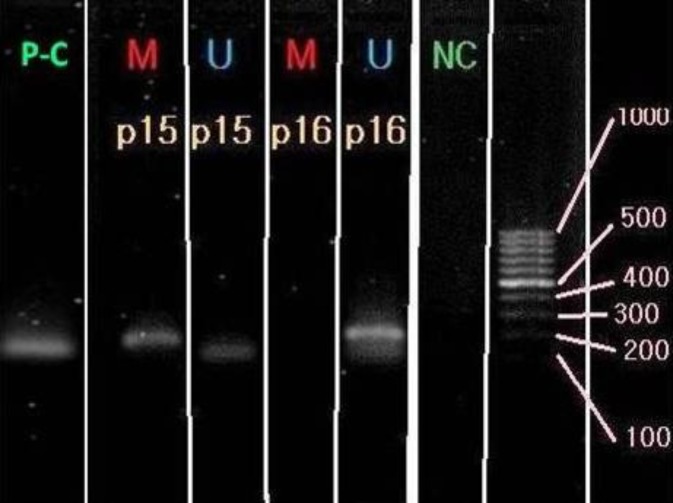
This Figure shows a 1.5% agarose gel in which the bands indicate PCR reaction products for non-methylated or methylated status of the genes under study in this research. NC and PC (Green) represent the negative and positive controls, respectively. The wells indicated by the letter M (Red) are related to the reactions in which primers specific to the methylated genes have been used, and the well marked with the letter U (Blue) is related to the reactions in which the target gene primers were used in unmethylated state

According to one of the best and most telling images recorded in predifferentiation stage, the results may be interpreted as follows: for gene P15, according to the bands observed in both reactions related to methylated and unmethylated primers, a partial methylation in the promoter of this gene is predictable at this point indicating its relative expression at this stage of the cell differentiation. In the other gene, P16, the band is only seen in the non-methylated primer related wells, which indicates the lack of methylation at this stage, hence, indicates the full expression of P15 gene (Figure 9). 

**Figure 10:**
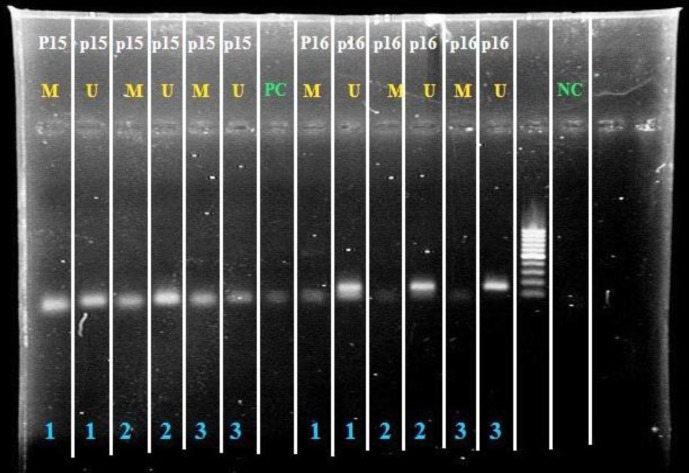
NC and PC (Green) represent the negative and positive controls, respectively. The wells indicated by letter M show the reactions in which primer of the gene in question has been used in methylated state, and the wells marked with the letter U (Blue) are related to the reactions in which the target gene primers were used in unmethylated state. Rows one, two and three (blue) correspond to three different annealing temperatures

Figure 10 shows MSP reaction results in post differentiation stage. According to the Figure 10, there are visible products in both reactions of the methylated and unmethylated primers for P15 and P16 genes indicating partial methylation in promoter and relative expression in post differentiation stage.

## Discussion

Epigenetics is well established as one of the pathways for regulation expression of a group of genes without any changes in their sequence (18). Expression of genes in gene expression profile of the cell is regulated by principles such as methylation and micro-RNA diversity (15). Induction of the differentiation to various cell types depends on regulation in gene expression level and specific factors such as elements controlling cell proliferation and death (e.g. P15INK4b and P16INK4a). With respect to the existence of CG Islands in promoter regions of these factors, methylation may be one of the ways to control their expression in the cell. Lack of the abovementioned elements is primarily observed in hematopoietic tissue malignancies, indicating the effective role of both factors in hematopoiesis control. Control of cell cycle in G1 Point in hematopoietic cells is among the important roles of P16 INK 4a and P15 INK 4b, respectively known as the P16 and P15 (9-13). Considering gene Expression profile of erythroid precursors, it should be mentioned that this profile is different from lymphoid precursors, and is similar to myeloid precursors. However, the genes expressed in erythroid precursors are mostly related to the immune responses, cell metabolism and lineage differentiation induction, while the genes expressed in primitive hematopoietic cells are associated with signaling and control of cell proliferation and death (23). In several studies, the expression mechanisms of erythroid-specific genes have been partly mentioned. A successful case of these investigations was the study by Charnay in 1984, explaining the expression mechanisms of beta and alpha globins. Accordingly, insertion of elements inducing erythroid differentiation in a specific sequence of GATAAG in 5’ region of beta and alpha globin gene promoter as well as a number of other erythroid-specific genes has been introduced as a major pathway to control the expression of these genes (24). In previous studies, it has been shown that P16 is highly expressed in hematopoietic stem cells, however, its expression is reduced during the differentiation to various lineages. The accuracy of this idea has been evaluated during the differentiation of cord blood hematopoietic cells to erythroid series. It should be noted that, based on previous studies, expression levels of P15 in hematopoietic cells are very low or negligible before the differentiation, but they are increased following myeloid and megakaryocyte differentiation. Expression results of P15 in hematopoietic stem cells in this study are not completely in agreement with previous studies. Expression pattern of P15 during erythroid differentiation has not been evaluated so far, and has been dealt with in the present study. In addition, the mechanisms controlling P15 and P16 genes expression in primitive hematopoietic cells have not been properly mentioned, and in this study, the role of epigenetics (methylation) as one of these mechanisms regulating the expression of these genes was investigated (25). Hematopoiesis has recently been accepted as a model for investigating epigenetic changes during lineage differentiation. Several studies have shown a downward trend for the pattern of methylation during the differentiation of primary hematopoietic cells to non-lymphoid lineages. Incidentally, methylation shows considerable increase during differentiation to lymphoid series (26). In addition, genes without methylation in the early stages of differentiation in hematopoietic cells remain demethylated after lineage differentiation. However, the methylated genes before the differentiation will become generally demehylated following the lineage differentiation (27). 

In the present study, the expression status of P15 and P16 was evaluated using Real-Time PCR in umbilical cord stem cells before and after the erythroid differentiation. The pattern of methylation in the promoter of these genes was assessed using MSP technique. Indeed, as it has been mentioned, MSP results for P15 gene before and after the differentiation showed partial methylation, while Real-Time results indicated relative expression of P15 in CD34+ hematopoietic cells, and a reduction of its expression after differentiation by 30% of the initial rate. It should be noted that with respect to an identical methylation patterns of P15 in both differentiation steps along with Real-Time results, downward regulation of the expression of this gene after differentiation may be due to other reasons in addition to the possible changes in methylation. On the other hand, in these cases it can be proposed that the methylation rate of the genes may have a role in changing pattern of the gene expression which is not detectable by MSP, as MSP can only report methylation or lack of methylation. For P16 gene, lack of methylation before differentiation is changed to partial methylation status after differentiation, and as expected, gene expression analysis results indicate reduced expression relative to the predifferentiation step. Therefore, it may be concluded that methylation changes may be a mechanism of downward regulation of P16 after differentiation, although hypermethylation of this gene was already mentioned in a variety of malignancies (10-12).

According to the results of the present study, expression of the P15 and P16 factors is not zero in primitive umbilical cord cells and erythroid precursors, indicating a defined role of both genes in metabolism of CD34+ stem cells before differentiation and in progenitors committed to erythroid lineage. Reduced expression of both factors after erythroid differentiation in presence of Mir 451 indicates a more important role of them in non-differentiation steps and in primitive cells of cord blood relative to erythroid precursors. 

## Conclusion

Taken together, our evidence suggests that specific predifferentiation expression of P15INK4b and P16INK4a genes along with reduction in their expression after erythroid differentiation indicated a more important role for these two genes in biology of CD34+ cells in primary stages and before differentiation. In addition, given that both genes are capable of epigenetic modifications due to structure of their promoters. Therefore, present study is the primary step in this field and further studies would be necessary. 
